# Supplementary material for: Protocol for a sequential, prospective meta-analysis to describe coronavirus disease 2019 (COVID-19) in the pregnancy and postpartum periods
Source: PLoS One. 2022 Jun 16;17(6):e0270150. doi: 10.1371/journal.pone.0270150 (PMC9202913; doi:10.1371/journal.pone.0270150)
Supplement: S2 File — (PDF) [file pone.0270150.s003.pdf]

Global Data Harmonization
Data Modules and Core Questions / Variables for Pregnancy & Perinatal COVID-19 Registries or Cohorts

(Last Updated November 8, 2020)

Prepared by Emily R. Smith[1] (EmilySmith@GWU.edu) and Siran He[1], in collaboration with Yalda Afshar[2] and Valerie Flaherman[3]

Updated by Emily R. Smith[1], Siran He[1], Erin Oakley[1], Mollie Wood[4]

[1]Department of Global Health, Milken Institute School of Public Health at The George Washington University, Washington, D.C., U.S.A. [2]Department of Obstetrics and Gynecology, University of California, Los Angeles, [3]Department of Pediatrics, University of California, San Francisco, [4]Cincinnati College of Medicine, Cincinnati

Objective: Our objective is to provide a harmonized set of high priority, core questions / variables for pregnancy and perinatal COVID-19 registry or cohort studies. These are not intended to be a survey or case report form. Each study has or will develop local protocols and data collection forms. However, each study will ideally collect the data outlined here. This will enable future collaborations to answer high priority questions or to pool data where studies investigators are willing and able.

Harmonization Process: We developed the draft data modules and questions based on a proposed set of questions from the PRIORITY study. We also reviewed and included questions from the data collection forms developed by the World Health Organization (WHO) and the U.S. Center for Disease Control (CDC). We requested feedback via a survey and by email from the >50 participants of the bi-monthly "Perinatal COVID-19 Global Gathering". The current data modules reflect feedback and general consensus among survey respondents.

Which studies should harmonize data? Any registry or cohort study collecting data regarding pregnant or postpartum people suspected or confirmed to have COVID-19 (as well as their fetus / infant) should consider collecting harmonized data, whether or not they are population-based. The studies may collect data from health records, by directly questioning the participant, or both. Differences in the way participants are sampled/recruited or the way data is collected can be reconciled when specific data analyses are planned.

Participant Inclusion Criteria: Participants enrolled in the study should meet the following inclusion criteria:

- Pregnant person, or person who was pregnant within the past 6 weeks *(Note: 42 days/ 6weeks reflects the postpartum period / maternal mortality definition);*
- diagnosed with (or suspected to be infected with) COVID-19;
- provides informed consent.

What is a Data Module: We define a "Data Module" as a group of questions (variables) that: 1) are thematically related; 2) are asked at the same time and with the same frequency; 3) AND refer to EITHER mother or baby (not both). These modules are organized for the purpose of prioritizing variables and themes, and do NOT reflect the order in which they should/would appear in actual data collection forms.

Updates: Updates to the data modules in August and September 2020 reflect efforts to harmonize these modules with updated World Health Organization (WHO) case definitions and ongoing multi-site study protocols developed by WHO. Additions are highlight in yellow. Questions that have been removed are crossed out.

Core Data Modules

**Module**	**Research Objective**
**Module 1**: Maternal COVID-19 Information<br>*(n=14 questions)*	To evaluate the clinical presentation and natural history of disease for women infected with (or suspected to be infected with) COVID-19 who are pregnant or have been pregnant within the last 6 weeks / 42 days. *(e.g., symptoms, testing, treatment, clinical course)*
**Module 2**: Pregnancy Status & Pregnancy-Related Morbidity<br>*(n=14 questions)*	To confirm pregnancy status and to document pregnancy-related basic information among women infected with COVID-19<br>*(e.g., due date, singleton/multiple pregnancy, etc.)*
**Module 3:** Pregnancy Outcomes<br>*(n=5 questions)*	To document the endpoint of the registered pregnancy among women infected with COVID-19<br>*(e.g., abortion, stillbirth, live birth, etc.)*
**Module 4:** Birth Characteristics<br>*(n=15 questions)*	To document various characteristics related to live birth.<br>*(e.g., place of birth, birthweight, gestational age, etc. Information about the infant, if recorded during/right after delivery, will be in this module)*
**Module 5:** Infant Morbidity & Mortality<br>*(n=8 questions)*	To evaluate infant outcomes among those born to women who have had COVID-19 (if live birth)<br>*(e.g., COVID-like symptoms up till 12mo, etc.)*
**Module 6**: Core socio-demographic information<br>*(n=6 questions)*	To identify high-risk subgroups with increased pregnancy, delivery, and infant adverse outcomes that are potentially associated with COVID-19
**Module 7:** Biospecimens and Diagnostic Testing<br>*(n=unlimited responses based on collected samples)*	To clearly document all biospecimens collected related to COVID-19 in a pregnancy and delivery and diagnostic testing for COVID-19 completed for the mother and infant, with a focus on identifying any instances of vertical transmission of COVID-19.

Time intervals (e.g. gestational age, time from symptom onset to testing) should be calculated directly from dates where possible. **The dates (and ages) recorded in these modules include:**

- Module 1-Q1: Date of onset of COVID-like symptoms
- Module 1-Q3: Date of COVID diagnosis
- Module 1-Q8: Date of hospital admission
- Module 1-Q13: Date of ICU admission / critical care receipt
- Module 2-Q1: Date of study/registry enrollment
- Module 2-Q4: Expected date of delivery
- Module 3-Q1: Date of pregnancy outcome
- Module 3-Q4: Date of maternal death
- Module 4-Q1: Date of birth & time of birth
    - Same as Module 3-Q1 (if live birth)
- Module 4-Q6: Infant gestational age estimation
    - Note this question asks about estimation method, which is important in order to address measurement error in analysis stage
- Module 4-Q14: Date of facility discharge after birth (for infant)
- Module 5-Q2: Date of neonatal death
- Module 5-Q4: Date of neonatal COVID-19 signs/symptoms
- Module 7-Q2a: Collection date of maternal biospecimen(s)
- Module 7-Q4a: Collection date of biospecimen(s) at delivery
- Module 7-Q6a: Collection date of neonatal/infant biospecimen(s)

Module 1: Maternal COVID-19 Information

What: COVID symptoms, testing, treatment, clinical course
When: At regular interval until disease resolution or chart abstraction

Questionnaire-based data collection	Medical record data extraction
Q1. Date of onset of COVID-like symptoms (DD:MM:YY)	Q1. Extract from medical record:<br>● First date of COVID-like symptoms (DD:MM:YY)
Q2. Have you been diagnosed with COVID-19?<br>● Yes, laboratory confirmed COVID-19<br>● Yes, probable COVID-19 case (see 2a)<br>● Suspected COVID-19 case (see 2b)<br>● No, COVID-19 negative	Q2. Extract from medical record:<br>● COVID-19 diagnosis notes:<br>○ Laboratory Confirmed<br>○ Probable COVID-19 case (see 2a)<br>○ Suspected COVID-19 case (see 2b)<br>○ No, COVID-19 negative
Q2a. If diagnosed as a probable COVID-19 case, by which criteria where you diagnosed?<br>• A patient who meets clinical criteria above AND is a contact of a probable or confirmed case, or epidemiologically linked to a cluster with at least one confirmed case.	Q2a. If diagnosed as a probable COVID-19 case, by which criteria was diagnosis made?<br>• A patient who meets clinical criteria above AND is a contact of a probable or confirmed case, or epidemiologically linked to a cluster with at least one confirmed case.

<table>
<tr><td>A suspect case with chest imaging showing findings suggestive of COVID-19 disease
A person with recent onset of anosmia (loss of smell) or ageusia (loss of taste) in the absence of any other identified cause.
Death, not otherwise explained, in an adult with respiratory distress preceding death AND was a contact of a probable or confirmed case or epidemiologically linked to a cluster with at least one confirmed case.</td><td>A suspect case with chest imaging showing findings suggestive of COVID-19 disease
A person with recent onset of anosmia (loss of smell) or ageusia (loss of taste) in the absence of any other identified cause.
Death, not otherwise explained, in an adult with respiratory distress preceding death AND was a contact of a probable or confirmed case or epidemiologically linked to a cluster with at least one confirmed case.</td></tr>
<tr><td>Q2b. If diagnosed as a probable COVID-19 case, by which criteria where you diagnosed?
A person who meets the clinical AND epidemiological criteria by WHO
A patient with severe acute respiratory illness</td><td>Q2b. If diagnosed as a probable COVID-19 case, by which criteria was diagnosis made?
A person who meets the clinical AND epidemiological criteria by WHO
A patient with severe acute respiratory illness</td></tr>
<tr><td>Q3. If answered “Yes” to Q2:
Date of COVID-19 diagnosis (DD:MM:YY)</td><td>Q3. Extract from medical record:
Date of COVID-19 diagnosis, or Date PUI status was documented (DD:MM:YY)</td></tr>
<tr><td>Q4. What symptoms did you have that led you to be tested or suspected of Coronavirus/COVID19? (Check all that apply)
Fever
Cough
Shortness of breath
Dizziness or fainting
Body aches
Runny nose
Sore throat
Loss of sense of smell
Loss of sense of taste
Sneezing
Fatigue
Nausea
Vomiting
Diarrhea
Headache
Other symptoms (please specify)
None / Asymptomatic</td><td>Q4. Extract from medical record:
All symptoms listed in the record that are related to COVID investigation / diagnosis</td></tr>
<tr><td>Q5. Do you work in healthcare or provide direct patient care?
Yes
No
Other, please specify</td><td>Q5. Extract from medical record:
If occupation data is available,
Note down “Y” for healthcare/direct patient care,
“N” for other occupations,
“NA” for unknown</td></tr>
<tr><td>Q6. Have you received any medication for the treatment of COVID-19 (e.g. anti-viral, immunomodulators, convalescent plasma, IL6 mAb, other)
Yes, I have
No, I have not
Maybe / uncertain
Other, please specify</td><td>Q6. Extract from medical record:
Has the patient been given any medication for the treatment of COVID-19
Yes
No
Other, please specify</td></tr>
</table>

Q7. If answered “Yes” to Q6: if possible, document more details about the medication:<br>● Type / Name<br>● Dose<br>● Duration<br>● Indications<br>● Clinical trial registration	Q7. Extract from medical record:<br>● Details about COVID-19 treatment regime, including medications, dose, duration, clinical trial inclusion, etc.
Q8. Were you ever admitted to the hospital for COVID-19<br>• Yes<br>• No<br>• Unknown	Q8. Extract from medical record: Ever admitted to the hospital for COVID-19<br>• Yes<br>• No<br>Unknown
Q9. If answer “Yes” to Q8”: What was the date of hospital admission? (DD:MM:YY)	Q9. Extract from medical record: What was the date of hospital admission? (DD:MM:YY)
Q10. If answer “Yes” to Q8”: What was the date of hospital discharge? (DD:MM:YY) (in the case of death, enter date of death).	Q10. If answer “Yes” to Q8”: Extract from medical record: What was the date of hospital discharge? (DD:MM:YY) (in the case of death, enter date of death).
Q11. If answer “Yes” to Q8”: what was the respiratory status of the patient while hospitalized?<br>• Self ventilating in room air<br>• Self ventilating with oxygen support<br>• Non-invasive respiratory support (CPAP, NIV)<br>• Mechanically ventilated, intubation<br>• Unknown	Q11. If answer “Yes” to Q8”: Extract from medical record: what was the respiratory status of the patient while hospitalized?<br>• Self ventilating in room air<br>• Self ventilating with oxygen support<br>• Non-invasive respiratory support (CPAP, NIV)<br>• Mechanically ventilated, intubation<br>• Unknown
Q12. If answer “Yes” to Q8”: Was the mother admitted to an intensive care unit (ICU) or administered critical care for COVID-19?	Q12. If answer “Yes” to Q8”: Extract from medical record: Was the patient admitted to an intensive care unit (ICU) or administered critical care for COVID-19?
Q13. If answer “Yes” to Q12”: What was the date of ICU admission / critical care? (DD:MM:YY)	Q13. If answer “Yes” to Q12”: Extract from medical record: What was the date of ICU admission / critical care? (DD:MM:YY)
Q14. If answer “Yes” to Q8”: What was the date of ICU discharge / end of critical care? (DD:MM:YY) (in the case of death, enter date of death).	Q14. If answer “Yes” to Q8”: Extract from medical record: What was the date of ICU discharge / end of critical care? (DD:MM:YY) (in the case of death, enter date of death).

Module 2: Pregnancy Status & Pregnancy-Related Morbidity

What: Pregnancy registration information and non-COVID morbidity among participants
When: At pregnancy registration

Questionnaire-based data collection	Medical record data extraction
Q1. Date of study enrolment (DD:MM:YY)	Q1. Extract from medical record:

	Date of study enrolment (DD:MM:YY)
Q2. Status upon enrolment<br>● Pregnant (confirmed by healthcare provider), not in labor<br>● Pregnant (not confirmed by healthcare provider), not in labor<br>● Pregnant in labor<br>● Postpartum [days] --> if yes, breastfeeding Y/N<br>● Post-abortion, miscarriage	Q2. Extract from medical record:<br>● Pregnancy status:<br>○ Pregnant (confirmed by healthcare provider) not in labor<br>○ Pregnant in labor<br>○ Postpartum [days] --> if yes, breastfeeding Y/N<br>○ Post-abortion, miscarriage
Q3. If answered "postpartum" in Q2: are you breastfeeding?<br>● Yes<br>● No	Q3. Extract from medical record:<br>● Breastfeeding status for postpartum women
Q4. What is your due date (expected date of delivery)?<br>● DD/MM/YYY = Date<br>● 08/08/1908 = Unknown<br>● 09/09/1909=Not applicable	Q4. Extract from medical record:<br>● Gestational age at enrollment
Q5. If answered "Yes" to Q4:<br>● What is the method of due date assessment?	Q5. Extract from medical record:<br>● Method of gestational age assessment
Q6. Number of fetuses:<br>● Singleton<br>● Twins<br>● Triplet<br>● Higher order	Q6. Extract from medical record:<br>● Number of fetuses
Q7. (Pre-pregnancy) BMI (can document BMI or weight and height):<br>● Weight (Kg)<br>● Height (m)	Q7. Extract from medical record:<br>● Pre-pregnancy weight and height (if available, be sure to note down units)
Q8. (At the time of registration) BMI (can document BMI or weight and height):<br>● Weight (Kg)<br>● Height (m)	Q8. Extract from medical record:<br>● At the time of clinical visit, weight and height (if available, note down units)
Q9. Gravidity: Is this your first pregnancy?<br>● Yes<br>● No	Q9. Extract from medical record:<br>● Gravidity information
Q10. If answered "No" to Q9:<br>● How many times have you been pregnant?	Q10. NA
Q11. Parity: Is this your first child birth?<br>● Yes<br>● No	Q11. Extract from medical record:<br>● Parity information
Q12. If answered "Not" to Q11:<br>● How many live births have you had previously?	Q12. NA
Q13. Has a doctor or other healthcare provider told you that you have any of the following conditions before you were pregnant? (check all that apply) *(local sites should*	Q13. Extract from medical record:<br>● List all pregnancy-related conditions documented in the record

describe conditions in a way that women will understand and self-report)<br>● Asthma<br>● Obesity<br>● Sleep apnea<br>● Anemia (Hb < 11g/dL per WHO)<br>● Chronic high blood pressure (hypertension)<br>● Thyroid disease<br>● Immune suppression (due to underlying disease or meds)<br>● Neurological disease<br>● Chronic lung disease (excluding asthma)<br>● Autoimmune disease<br>● Cardiovascular disease (excluding hypertension)<br>● Other, please note	
Q14. Has a doctor or other healthcare provider told you that you have any of the following conditions during this pregnancy? (check all that apply) *(local sites should describe conditions in a way that women will understand and self-report)*<br>● Hypertensive disease of pregnancy (including preeclampsia/eclampsia)<br>● Hyperemesis<br>● Intrauterine growth restriction<br>● Abnormal placentation (placental previa/accreta/percreta)<br>● Placental abruption<br>● Bacterial infection prior to hospital visit<br>● Preterm contractions (not in labor)<br>● Preterm labor<br>● Preterm rupture of membranes<br>● Haemorrhage<br>● If haemorrhage, which type: antepartum/intrapartum; Postpartum; Abortion-related<br>● Embolic disease<br>● Anesthetic complications	Q14. Extract from medical record:<br>● List all pregnancy-related conditions documented in the record

Module 3: Pregnancy Outcomes

What: Information about how the pregnancy ended; maternal mortality will be recorded in this module
When: Once, after pregnancy outcome is known

Questionnaire-based data collection	Medical record data extraction
Q1. Date of pregnancy outcome (DD:MM:YY) *[Allows calculation of gestational age (in weeks) when pregnancy ended]*	Q1. Extract from medical record:<br>● Date of pregnancy outcome (DD:MM:YY)
Q2. Please indicate the end point of this pregnancy:<br>● Live birth	Q2. Extract from medical record:<br>● The end point of this pregnancy (note down

● Stillbirth<br>● SAB (spontaneous abortion) → expectant, medical management, D&C/E (3 choices)<br>● TAB (therapeutic abortion) → expectant, medical management, D&C/E (3 choices)	based on the format in the record, such as live birth, stillbirth, etc.)
Q3. Maternal death?<br>● Yes<br>● No<br>● Unknown	Q3. Extract from medical record:<br>● If maternal death occurred (yes/no/unknown)
Q4. If answered "Yes" for Q3:<br>● Date of maternal death? (DD:MM:YY)	Q4. Extract from medical record:<br>● If maternal death, date of death (DD:MM:YY)
Q5. If answered "Yes" for Q3:<br>● Cause of death<br>o COVID-19<br>o Obstetric hemorrhage<br>o Hypertensive disorder (including preeclampsia and eclampsia)<br>o Pregnancy-related infection<br>o Abortion/ectopic pregnancy<br>o Other direct cause (obstetric complications)<br>o Indirect cause (pre-existing medical condition exacerbated by pregnancy)<br>o Coincidental cause (e.g. motor vehicle cause, accidental injury, assault)<br>o Unknown	Q6. Extract from medical record:<br>● If maternal death, cause of maternal death (COVID or other causes, document as appeared in medical record)

Module 4: Birth Characteristics

What: Information related to labor and delivery/birth and other data collected on the day of birth
When: To be asked on or soon after the day of birth, at enrollment if enrollment occurs postpartum, or directly extracted from chart

Questionnaire-based data collection	Medical record data extraction
Q1. Date (and time, if available) of birth (DD:MM:YY) (HH:MM)	Q1. Extract from medical record:<br>● Date of birth (and time of birth, if available) (DD:MM:YY) (HH:MM)
Q2. Where was the infant born? *<br>● Home<br>● Birthing facility/hospital<br>● Other location, please specify<br><br>** If your registry collects only hospital-based births, please record "facility/hospital" for all records*	Q2. Extract from medical record:<br>● Location of birth:<br>○ Hospital (indicate which hospital)<br>○ Other (specify location, if delivery did not occur at this facility)<br>○ "NA" if unknown

Q3. Mode of delivery?<br>● Spontaneous vaginal<br>● Operative vaginal - forceps or vacuum<br>● Cesarean section (scheduled)<br>● Cesarean section (intrapartum)<br>● Unknown	Q3. Extract from medical record:<br>● Mode of delivery exactly as documented in the record
Q4a. What was the infant's weight at birth (grams)?	Q4a. Extract from medical record:<br>● Infant weight at birth (grams)
Q4b. What was the infant's length at birth (cm)?	Q4b. Extract from medical record:<br>• Infant length at birth (cm)
Q4c. What was the head circumference at birth (cm)?	Q4c. Extract from medical record:<br>• Infant head circumference at birth (cm)
Q5. Has the infant's gestational age been estimated?<br>● Yes<br>● No	Q5. NA
Q6. If answered "Yes" to Q6:<br>● What was the estimated gestational age at birth (weeks)	Q6. Extract from medical record:<br>● Gestational age of infant
Q7. If answered "Yes to Q6:<br>● What method was used to determine gestational age?<br>○ Ultrasound<br>○ Estimated date of delivery based on last menstrual period (EDD)<br>○ Ballard/Dubowitz;<br>○ Other, please specify	Q7. Extract from medical record:<br>● Estimation method exactly as documented in the record
Q8. On the day of birth, did your infant have any difficulty breathing?<br>● Yes<br>● No<br>● Maybe / Uncertain	Q8. Extract from medical record:<br>● On the day of birth, did the infant have any difficulty breathing, as documented in the record
Q9. At the time of birth, was your infant admitted to the neonatal intensive care unit or the special care unit?<br>● Yes<br>● No	Q9. Extract from medical record:<br>● At the time of birth, was the infant admitted to the neonatal intensive care unit/special care unit (Y/N/NA)
Q10. At the time of birth, did your infant receive oxygen?<br>● Yes<br>● No	Q10. Extract from medical record:<br>● At the time of birth, did the infant receive oxygen? (Y/N/NA)
Q11. What type of resuscitation was provided at delivery?<br>● None<br>● Warm/dry/stimulation<br>● Blow by oxygen<br>● Continuous positive airway pressure<br>● Positive pressure ventilation<br>● Intubation<br>● Chest compressions<br>● Surfactant	Q11. Extract from medical record:<br>● Resuscitation? (Y/N/NA)<br>● If yes → type of resuscitation, as documented in the record

• Unknown	
Q12. Did the infant breastfeed or receive any breast milk on the day of birth?<br>• Yes<br>• No<br>• Maybe / Uncertain	Q12. Extract from medical record:<br>• Breastfeeding or breast milk on the day of birth (Y/N/NA)
Q13: Was the newborn isolated away from mother in another area in hospital (postnatal ward, special care nursery, NICU or special ward)?	Q13: Extract from medical record:<br>• Was the newborn isolated away from mother in another area in hospital (postnatal ward, special care nursery, NICU or special ward)?
Q14. Date of infant discharge from labor and delivery event (DD:MM:YY)	Q14. Extract from medical record:<br>• Date of infant discharge (DD:MM:YY)
Q15. Newborn outcome at discharge:<br>• Expired<br>• Home<br>• Transfer to another acute care facility due to clinical needs<br>• Transfer to a chronic care facility	Q15. Extract from medical record:<br>• Newborn outcomes at discharge as documented in the record

Module 5: Infant Mortality and Morbidity

What: Information related to infant health, particularly related to COVID-19
When: At regular interval until up to 12 months after birth (or until chart extraction)

**Questionnaire-based data collection**	**Medical record data extraction**
Q1. Neonatal/infant death?<br>• Yes<br>• No	Q1. Extract from medical record:<br>• Neonatal/infant death? (Y/N/NA)
Q2. If answered "Yes" to Q1:<br>• Date-time of neonatal/infant death? (DD:MM:YY) (HH:MM)	Q2. Extract from medical record:<br>• If neonatal/infant death, date-time of death (DD:MM:YY) (HH:MM)
Q3. If answered "Yes" to Q1:<br>• Cause of death?<br>○ COVID-19<br>○ Preterm/low birth weight<br>○ Birth asphyxia<br>○ Infection<br>○ Birth trauma<br>○ Congenital/birth defects<br>○ Unknown<br>○ Other, please specify	Q3. Extract from medical record:<br>• If neonatal/infant death, cause of death (COVID or other causes, document as appeared in medical record)
Q4. Was the infant documented by clinicians to have any of the following symptoms of COVID-19?<br>- Fever (T>37.5)<br>- Respiratory distress	Q4. Extract from medical record:<br>• Was the infant documented by clinicians to have any of the symptoms of COVID-19? (document exactly as in the record)

- Cough<br>- Nasal congestion/runny nose<br>- Vomiting<br>- Diarrhea<br>- Lethargy<br>- Rapid heart rate (>160 bpm) (record bpm if available)<br>- Seizure<br>- Paralysis<br>- Hypotonia (floppiness)<br>- Hypertonia or Stiffness or spasticity of limbs<br>- Other neurological signs<br>- Rash<br>- Oedema<br>- Eye redness/conjunctivitis<br>- Other condition<br><br>*If yes, date of onset?*	
Q5. Was the baby tested for COVID-19?<br>● Yes<br>● No<br>● Maybe / Uncertain	Q5. Extract from medical record:<br>● Any COVID-19 test for the infant? (Y/N)<br><br>*If answered "Yes" go to Module 7 to record details.*
Q6. If answered "Yes" to Q5:<br>● What tests were conducted?<br>○ Biospecimen: viral, antibody, or antigen test (record details in Module 7)<br>○ Chest image or X-ray<br>○ Lung ultrasound<br>○ Echcardiogram<br>○ Cerebral ultrasound<br>○ Abdominal ultrasound<br>○ Other, please specify<br><br>*If answered "Yes" to "Biospecimen" - go to Module 7 to record details.*	Q6. Extract from medical record:<br>● Which tests were conducted
Q7. Has your infant breastfed or received any breast milk in the last 24 hours?<br>● Yes<br>● No<br>● Unknown	Q7. Extract from medical record:<br>● Has the infant been breastfed or received any breast milk in the past 24 hours (Y/N/NA)?<br>● If information is available for longer periods, note it down as well
Q8. Any congenital anomalies?<br>● Neural tube defects<br>● Microcephaly<br>● Congenital malformations of ear<br>● Congenital heart defects<br>● Orofacial clefts<br>● Congenital malformations of digestive system<br>● Congenital malformations of genital organs<br>● Abdominal wall defects<br>● Chromosomal abnormalities<br>● Reduction defects of upper and lower limbs	Q8. Extract from medical record:<br>● Any congenital anomalies in the record? (Note it down exactly as in the record)

• Talipes equinovarus/clubfoot<br>• Other, please specify	

Module 6: Core Sociodemographic Information

What: Socio-demographic information about the study participant
When: Once at the beginning of the study (maternal information); Once after birth (for infant information)

Questionnaire-based data collection	Medical record data extraction
Q1. Country | Site ID	Q1. Indicate the site / facility
Q2. Age of the mother (years)	Q2. Extract from medical record:<br>• Mother's age (years), or<br>• Mother's date of birth (DD:MM:YY)
Q3. Sex of the child (if applicable)<br>• Male<br>• Female<br>• Ambiguous<br>• Unknown	Q3. Extract from medical record:<br>• Sex of the child
Q4. Race/Ethnicity of the mother *<br><br>** Each site to define appropriate categories for their context*	Q4. Extract from medical record:<br>• Mother's race/ethnicity (if available)
Q5. Race/Ethnicity of the child (if applicable) *<br><br>** Each site to define appropriate categories for their context*	Q5. Extract from medical record:<br>• Child's race/ethnicity (if available)
Q6. Years/level of education completed by the mother (check highest level completed)<br>• No formal education<br>• Some primary education<br>• Primary education completed<br>• Secondary education completed<br>• University/College completed<br>• Graduate education /Terminal degree completed	Q6. Extract from medical record:<br>• Mother's education level (if available)

Module 7: Biospecimens and Diagnostic Testing

What: Biological specimens collected from mother and infant during pregnancy, at delivery, and postpartum, with a focus on indicators of vertical transmission of COVID-19.
When: Maternal biospecimens collected during the pregnancy, at the time of delivery, or after delivery, as well as biospecimens collected during delivery and from the infant at birth and during the first 6 weeks of life.

Questionnaire-based data collection	Medical record data extraction
Q1. Were any biospecimens collected for COVID-19 diagnostic testing from the mother during pregnancy, at the time of delivery, or in the postpartum period?	Q1. Extract from medical record:<br>• Any biospecimen/diagnostic testing for the mother? (Y/N)
Q2. If answered "Yes" to Q1: → Proceed to maternal biospecimen table below	See Q2 Maternal Biospecimen Table below
Q3. Were any biospecimens collected for COVID-19 diagnostic testing at the time of the delivery?	Q3. Extract from medical record:<br>● Any biospecimen/diagnostic testing at delivery? (Y/N)
Q4. If answered "Yes" to Q3: → Proceed to delivery-related biospecimen table below	See Q3 Delivery-Related Biospecimen Table below
Q5. Were any biospecimens collected for COVID-19 diagnostic testing from the infant?	Q5. Extract from medical record:<br>Any biospecimen/diagnostic testing for the infant? (Y/N)
Q6. If answered "Yes" to Q5: → Proceed to infant biospecimen table below	See Q3 Infant Biospecimen Table below

Q2. Maternal Biospecimen Table

Sample Number (as needed)	2a. Date of Biospecimen Collection	2b. Type of Biospecimen	2c. Type of Testing Conducted	2d. Qualitative Results	2e. Quantitative Result (e.g., viral load)	2f. Specify units for Quantitative Results
SM1.	DD:MM:YY	• Nasopharyngeal swab<br>• Vaginal swab<br>• Feces/rectal swab<br>• Maternal blood<br>• Breast milk<br>• Pregnancy tissue (in the case of fetal demise/induced abortion)<br>• Other, specify	• Viral PCR<br>• IgM<br>• IgG<br>• Other, specify	• Positive<br>• Negative<br>• Other, specify	___	________<br>________
SM2.						
SM3.						
SM4.						

Q4. Delivery-Related Biospecimen Table

Sample Number (as needed)	4a. Date of Biospecimen Collection	4b. Type of Biospecimen	4c. Type of Testing Conducted	4d. Qualitative Results	4e. Quantitative Result (e.g., viral load)	4f. Specify units for Quantitative Results
SD1.	DD:MM:YY	• Amniotic fluid<br>• Placental swab (any side)<br>• Placental swab (fetal side)<br>• Cord blood<br>• Other, specify	• Viral PCR<br>• IgM<br>• IgG<br>• Other, specify	• Positive<br>• Negative<br>• Other, specify	___	________<br>________
SD2.						
SD3.						

SD4.						

Q6. Infant Biospecimen Table

Sample Number (as needed)	6a. Date of Biospecimen Collection	6b. Type of Biospecimen	6c. Type of Testing Conducted	6d. Qualitative Results	6e. Quantitative Result (e.g., viral load)	6f. Specify units for Quantitative Results
SI1.	DD:MM:YY	• Nasopharyngeal swab<br>• Feces/rectal swab<br>• Gastric swab<br>• Neonatal peripheral blood<br>• Other, specify	• Viral PCR<br>• IgM<br>• IgG<br>• Other, specify	• Positive<br>• Negative<br>• Other, specify	___	________<br>________
SI2.						
SI3.						
SI4.						
